# An evaluation of the impact of large-scale interventions to raise public awareness of a lung cancer symptom

**DOI:** 10.1038/bjc.2014.596

**Published:** 2014-12-02

**Authors:** L Ironmonger, E Ohuma, N Ormiston-Smith, C Gildea, C S Thomson, M D Peake

**Affiliations:** 1 Statistical Information Team, Cancer Research UK, Angel Building, 407 St John Street, London EC1V 4AD, UK; 2 Knowledge and Intelligence Team (East Midlands), Public Health England, Sheffield S10 3TG, UK; 3 Information Services Division, NHS National Services Scotland, Edinburgh EH12 9EB, UK; 4 Department of Respiratory Medicine, Glenfield Hospital, Leicester LE3 9QP, UK; 5 National Cancer Intelligence Network, Public Health England, Wellington House, London SE1 8UG, UK; 6 Royal College of Physicians, London NW1 4LE, UK

**Keywords:** lung cancer, persistent cough, symptoms, awareness, health campaign, stage

## Abstract

**Introduction:**

Long-term lung cancer survival in England has improved little in recent years and is worse than many countries. The Department of Health funded a campaign to raise public awareness of persistent cough as a lung cancer symptom and encourage people with the symptom to visit their GP. This was piloted regionally within England before a nationwide rollout.

**Methods:**

To evaluate the campaign's impact, data were analysed for various metrics covering public awareness of symptoms and process measures, through to diagnosis, staging, treatment and 1-year survival (available for regional pilot only).

**Results:**

Compared with the same time in the previous year, there were significant increases in metrics including: public awareness of persistent cough as a lung cancer symptom; urgent GP referrals for suspected lung cancer; and lung cancers diagnosed. Most encouragingly, there was a 3.1 percentage point increase (*P*<0.001) in proportion of non-small cell lung cancer diagnosed at stage I and a 2.3 percentage point increase (*P*<0.001) in resections for patients seen during the national campaign, with no evidence these proportions changed during the control period (*P*=0.404, 0.425).

**Conclusions:**

To our knowledge, the data are the first to suggest a shift in stage distribution following an awareness campaign for lung cancer. It is possible a sustained increase in resections may lead to improved long-term survival.

Around 35 000 people were diagnosed with lung cancer in England in 2011 and 28 000 people died from the disease (Cancer Research UK, 2014). Despite advances in all aspects of care, the overall long-term survival from lung cancer has not improved greatly in recent years (Quaresma *et al*, 2014, in press). This is likely to be because, in its early stages, many cases of lung cancer are symptom free. Symptoms that do occur are often non-specific and do not immediately trigger action in patients or GPs. Therefore, by the time most patients reach specialist care they have locally advanced or metastatic disease, which is largely incurable with current treatments. Lung cancer survival in England is worse than many other countries (Coleman *et al*, 2011). This can be explained, at least in part, by a combination of late diagnosis and poorer stage-specific survival suggesting lower treatment rates (Walters *et al*, 2013), and higher rates of comorbidity (Imperatori *et al*, 2006). Further evidence for late diagnosis comes from a study comparing excess death rates for lung cancer 5 years from diagnosis in England, Norway and Sweden between 2001 and 2004 (Holmberg *et al*, 2010). Virtually all the excess deaths in England were confined to the first year after diagnosis. Since there is no screening programme for lung cancer in any of these three countries, a reasonable assumption is that patients with symptoms are being diagnosed and treated earlier in Sweden and Norway. In England around 40% of lung cancer patients first come to the attention of secondary care via an emergency admission and around 12% of such patients are alive 1 year after diagnosis, compared with 35% of those referred electively by their GPs (Elliss-Brookes *et al*, 2012). One estimate is that if England was to improve its lung cancer survival to match the best in Europe, around 1300 deaths at 5 years from diagnosis could be avoided annually (Abdel-Rahman *et al*, 2009).

Low cancer symptom awareness is likely to contribute to patient delays in presenting to medical professionals (Smith *et al*, 2009), and in turn contribute to later stage diagnosis. A systematic review by Austoker *et al* (2009) found limited evidence of the effectiveness of community-level interventions to promote cancer awareness with some evidence they can promote earlier stage at diagnosis, but only one study demonstrated a sustained effect of the campaign over several years. The review did not find any studies on lung cancer symptom awareness interventions.

However, a pilot community-based lung cancer symptom awareness intervention has since been published (Athey *et al*, 2012). The relatively small-scale project in the Doncaster area of England used a range of tools to raise public and primary care awareness of persistent cough as a sign of lung cancer. The project resulted in enhanced public recall of cough as an important symptom; behaviour change with more patients visiting their GP; a change in GP behaviour with a 20% increase in chest X-ray (CXR) requests; and an increase in lung cancer diagnoses. There was a suggestion that more patients were diagnosed with early-stage disease.

This suggestive evidence prompted the Department of Health (DH), as part of the National Awareness and Early Diagnosis Initiative (NAEDI), to fund a series of local pilot projects followed by a larger regional pilot, and subsequently a national campaign, aimed at raising public awareness of persistent cough as a symptom of lung cancer, targeting those aged 50 and over. There are many symptoms of lung cancer, none of which are highly specific for the disease (Shim *et al*, 2014), but in a number of studies cough emerges as the most common symptom, being recorded as a presenting symptom at the time of diagnosis in over half of cases (Hamilton *et al*, 2005; Smith *et al*, 2009). In their Referral Guidelines for Suspected Cancer, NICE identified cough persisting for more than 3 weeks as one of its major trigger symptoms (National Institute for Health and Care Excellence (NICE), 2005). There are no prospective, population-based studies examining the predictive value of symptoms or symptom clusters in lung cancer and for many reasons, not least of which is the huge size and cost of such a study, there is no prospect of such evidence becoming available. The DH took the pragmatic view that, while the symptom profile of lung cancer is obviously complex, the public ‘message' had to be kept simple, so the single symptom of cough persisting for 3 or more weeks was chosen as the basis of the regional and national campaigns. The primary hypothesis underlying the initiative was that promoting public awareness of persistent cough in people over 50, together with stressing the value of early presentation in terms of the potential for effective treatment, should result in a higher proportion of patients with lung cancer being diagnosed at a stage early enough to undergo potentially curative treatment.

## Materials and methods

The national campaign ran in England 8th May–30th June 2012, following the regional pilot, which ran in the Central TV region (mainly East and West Midlands) 10th October–13th November 2011 with remaining areas in England considered as the control area. The campaign used the ‘Be Clear on Cancer' brand with the strapline ‘Been coughing for 3 weeks or more? Tell your doctor'. The Supplementary Materials and Methods provides details of campaign materials.

### Data collection and analysis

To evaluate the impact of the national and regional campaigns, data were collected and analysed for the range of metrics outlined below, to represent the patient pathway from symptom awareness, through to diagnosis, stage, treatment and survival. Provided here is a summary of methods specific to the national campaign data, with the exception of the survival data that relate to the regional campaign since survival data are not yet available following the national campaign. More details of the methods, including details specific to the regional campaign data, are provided in the Supplementary Materials and Methods.

Data were obtained from a range of existing national data sets for the metrics where possible, otherwise bespoke data collection was required. For each metric, to establish whether there was a change following the campaign launch, data relating to the period during and/or immediately after the campaign were compared with data from a pre-campaign time period (most commonly the same time in the previous year where appropriate or available). For the regional campaign, changes in the pilot area were compared with changes in a control area. However, as no geographical control was available for the national campaign, a time period control was identified for metrics wherever possible. The time periods considered vary between metrics due to data availability. Our hypothesis is that changes in the controls represent the background trends and any changes around the campaign period greater than the controls may suggest a campaign impact. However, graphs of trends over the year of the campaign and previous 2 years were also produced for each metric, where possible, to observe any changes in the context of the longer-term trends.

To test the statistical significance of any changes between time periods or areas, changes in proportions were tested using the two-sample test of proportions, and changes in counts were tested using the likelihood ratio test of two counts. When there were significant changes for both the campaign and control periods (/area), Poisson regression models were used to test whether a change during the campaign period (/area) was significantly different from the change in the control. All statistical tests were carried out in Stata 11 (StataCorp, 2009).

#### Public awareness and perceived impact on GPs

Pre- and post-campaign random location quota surveys were undertaken by TNS BMRB (2014) to evaluate the impact of the campaign on measures including public awareness of symptoms of lung cancer for those aged 55+, with questions informed by the Cancer Awareness Measure (Stubbings *et al*, 2009). They also carried out surveys to assess GPs' views on numbers of patients presenting with symptoms of lung cancer, numbers of suspected lung cancer referrals made, as well as GPs' views on the campaign's communications.

Results from the national campaign awareness surveys include nested pilot region results, which can be considered as an assessment of the longer-term impact on awareness (around 6 and 8 months after the regional campaign), compared with the weeks immediately following the regional campaign (as assessed by the post-campaign survey).

#### Presentations to primary care

With a quota sample of 486 GP practices, healthcare IT specialists, Mayden (2014), used Read codes to provide data on numbers of patients presenting to GP practices with symptoms directly linked to the campaign (a cough) and selected control symptoms each week between March 2010 and May 2013. Numbers of visits over the 8 weeks around the campaign were compared with the same weeks in the previous year. For a control period, data for the 8 weeks before the campaign were compared with the equivalent period in the previous year. Numbers of attendances were adjusted for a 5-day week excluding public holidays (‘working days').

#### Urgent GP referrals for suspected lung cancer

Urgent GP referrals for suspected lung cancer were obtained from the National Cancer Waiting Times Monitoring Dataset provided by NHS England (2014a).

Monthly numbers of referrals in England were provided for January 2010 to November 2012. Numbers of referrals made during the months of the campaign and month directly following, May–July 2012, were compared with May–July 2011, referred to from here as the ‘campaign period'. A ‘control period' was selected as the 3 months before the campaign, February–April 2012, compared with February–April 2011. Data were adjusted for working days.

The proportion of urgent referrals for suspected lung cancer that resulted in a diagnosis of lung cancer (the conversion rate) was also provided.

#### CXRs and CT scans

Chest X-ray and chest with or without abdomen CT scan (CT) data were extracted from the Diagnostic Imaging Dataset (DID) provided by NHS England (2014b). The DID only includes data from April 2012, making comparisons with 2011 impossible; instead the number of CXRs and CTs from all referral pathways, and those following a GP referral specifically, in May–July 2012 were compared with April 2012, after adjustment for the number of organisations submitting data. GP-referred investigations were further adjusted for working days under the assumption that GPs only work these days; data for all investigations were adjusted for all days in the month, as these include referrals from pathways used on both working and non-working days. For a control, the same comparisons were made for 2013.

#### Presentation, stage at diagnosis and treatment in secondary care

The Health and Social Care Information Centre (HSCIC) provided data on the clinical impact of the campaign from the National Lung Cancer Audit (NLCA) database (HSCIC, 2013), following approval from the Healthcare Quality Improvement Partnership (HQIP, 2014).

Data were aggregated for all English NHS trusts involved in the care of lung cancer for confirmed lung cancer patients referred and first seen for suspected lung cancer between January 2010 and December 2012. Data during the campaign and control periods were analysed as described in the section ‘Urgent GP referrals for suspected lung cancer'. The following metrics were analysed.

- **Number of cases diagnosed.**
- **Stage at diagnosis:** For non-small cell lung cancer (NSCLC), data were available for stage at diagnosis (stage I, II, IIIA, IIIB, and IV) as defined by TNM seventh edition (Sobin *et al*, 2009). The changes in proportions diagnosed at each stage were tested with and without exclusion of cases with unknown stage (uncertain or not recorded). For small cell lung cancer (SCLC), proportions diagnosed with SCLC-limited stage and SCLC-extensive stage were also tested with and without exclusion of those with unknown stage.
- **First definitive treatment.**
- **Performance status:** Performance status was split into three groups: ‘0 and 1' ‘2' ‘3 and 4', which correspond to patients' likely fitness for treatment, and outcomes in terms of survival (with ‘0 and 1' being the best prognostic group).
- **NLCA source of referral.**
- **One-year survival:** Data are currently only available for analysing the impact of the regional campaign. The proportion of patients alive 1 year after diagnosis was compared for patients first seen in the pilot trusts in October–December 2011 with those seen in October–December 2010 and for the same periods for patients first seen in control trusts. Age-standardised 1-year crude survival was calculated using International Cancer Survival Standard weights (Corazziari *et al*, 2004).

## Results

### Public awareness and perceived impact on GPs

Public awareness of the target symptom increased after the campaign (Table 1). When survey participants were asked to name as many symptoms of lung cancer as possible (‘spontaneous awareness'), the proportion mentioning a cough increased from 54% pre-campaign to 65% post-campaign (*P*<0.001), with specific mentions of a persistent/prolonged cough increasing from 12% to 15% (*P*=0.048). When shown a list of lung cancer symptoms and asked how much of a lung cancer warning sign each was (‘prompted awareness'), a cough for 3 or more weeks was the symptom with the largest increase in recognition, rising from 18% pre-campaign to 33% after (*P*<0.001). Additionally, post-campaign awareness was higher among participants recognising at least one campaign advertisement than those saying they did not recognise any. For example, spontaneous awareness of cough was 69% in campaign recognisers compared with 48% in non-recognisers (*P*<0.001).

Similar trends were seen in the regional pilot area following the regional campaign (see Supplementary Table S1), for instance, prompted awareness of a cough for 3 or more weeks increased from 19% to 34% (*P*<0.001). For comparison, any changes in the control area were generally not significant. In the national campaign's pre-campaign survey, prompted awareness of a cough for 3 or more weeks had decreased for those in the pilot region since the post-regional campaign survey, but remained higher than for those in the control area (25% *vs* 16% *P*=0.002). Post-national campaign, prompted awareness was similar in both areas (33% *vs* 34%), and was also similar to post-campaign awareness in the regional area following the regional campaign (34%).

With the potential for the campaign's focus on one symptom to falsely reassure the public that other symptoms, in the absence of a cough, are not a sign of lung cancer, the impact on awareness of other lung cancer symptoms was also assessed. After the national campaign there was a small but significant decrease in the proportion spontaneously mentioning ‘coughing up blood' as a symptom of lung cancer (from 19% to 16% *P*=0.031), although there was no decrease in the proportion saying coughing up blood was definitely a warning sign (prompted awareness). Neither spontaneous nor prompted awareness for other lung cancer symptoms mentioned in the survey decreased after the national campaign (Table 1). Following the regional campaign, in the pilot area there was a significant fall in spontaneous mentions of chest pain (from 13% to 9% *P*=0.026), yet there was an increase in prompted awareness for this symptom (from 12% to 17% *P*=0.026).

Surveyed GPs' estimates of the numbers of patients presenting with a persistent cough in the past couple of months, and of referrals made for suspected lung cancer, increased following the national campaign (see Supplementary Table S2 for the national and Supplementary Table S3 for the regional campaign results). Additionally, over 80% of GPs (who had heard of the campaign) agreed that ‘it is important that advertising like this is shown'.

### Presentations to primary care

More people with a cough went to see their GP following the campaign launch (Table 2). Presentations of patients aged 50+ with a cough increased by 63% for the 8 weeks around the campaign compared with the same weeks in 2011 (adjusted for working days; *P*<0.001). This is equivalent to around three additional visits per practice per week (based on practices in the sample that had an average list size of around 7800 patients, while the England average was around 6800 for 2011/12 (HSCIC, 2012)). For comparison: for those aged 50+ the largest increase in presentations for the control symptoms was 5% for UTI (*P*=0.016); and just 5% more people presented with a cough during the 8 pre-campaign control weeks in 2012 compared with those weeks in 2011 (*P*<0.001). This suggests the increase following the campaign launch was over and above a small year on year increase in presentations (see also Supplementary Figure S1).

Across all ages, there was a 67% increase in patients visiting their GP with a cough (adjusted; *P*<0.001), equivalent to six additional visits per practice per week (data not shown). The age group with the highest actual increase in presentations during the campaign weeks was 60–69 year olds and the largest percentage increase was in 50–59 year olds (Supplementary Table S4).

The heightened increase in presentations appeared to continue for at least 8 weeks after the campaign ended (see Supplementary Figure S1). For the 8 weeks post-campaign, there were 46% more presentations in those aged 50+ compared with the same period in 2011 (*P*<0.001).

During the regional campaign (Supplementary Table S5), there was a 22% increase in presentations for a cough among those aged 50+ in the pilot area compared with the same time in the previous year (*P*<0.001). Data were not collected for the control area.

### Urgent GP referrals for suspected lung cancer

There was an increase in urgent GP referrals for suspected lung cancer (Table 3). The number of referrals increased by 31.8% for the campaign period (adjusted for working days; *P*<0.001), equivalent to 0.14 additional referrals per practice per month. Referrals increased by 3.8% for the pre-campaign control period (*P*=0.006) with evidence that this increase was significantly smaller than the increase for the campaign period (*P*<0.001), suggesting that the increase in referrals associated with the campaign period is elevated above a small year on year increase. An elevated increase appears to last until at least October 2012 (see Supplementary Figure S2A).

The conversion rate decreased by 2.5 percentage points for the campaign period (from 24.0% to 21.5% *P*<0.001), but the rate also dropped for the control period by 2.0 percentage points (from 24.3% to 22.3% *P*<0.001), with no evidence the decreases were significantly different (*P*=0.479). Supplementary Figure S2B also suggests that there was a general downward trend in conversion rates in 2012.

For the regional campaign (Supplementary Table S6), the number of urgent referrals similarly increased in the pilot area (27.6% increase; *P*<0.001) and it was also significantly higher (*P*<0.001) than the increase in the control area (7.9% *P*<0.001). The conversion rate did not significantly change in either area (data not shown).

### CXRs and CT scans

The number of CXRs following a GP referral increased following the campaign launch (Table 4). Specifically, the number of GP-referred CXRs increased by 18.6% in May–July 2012 compared with April 2012 with adjustment for working days (*P*<0.001). For comparison, the number of CXRs from all referrals increased by 7.3% comparing May–July with April 2012 when adjusting for total number of days (*P*<0.001). Also, as a control comparison, for the same period in 2013, GP-referred CXRs per working day decreased by 16.7% for May–July compared with April (*P*<0.001).

The number of GP-referred CTs per working day also increased, with an increase of 15.7% comparing May–July 2012 with April (from 0.6 to 0.7 per organisation per working day; *P*<0.001). For comparison with a control trend, in 2013 there was a 5.8% decrease for May–July compared with April (*P*=0.025).

### Presentation, stage at diagnosis and treatment in secondary care

#### Number of cases diagnosed

The number of lung cancers diagnosed increased after the campaign launch (Table 5). There was a 9.1% increase in lung cancers diagnosed during the campaign period (adjusted for working days; *P*<0.001), while there was a small non-significant 1.5% increase during the control period (*P*=0.373). Similar results were seen in the regional campaign (Supplementary Table S7).

Supplementary Figure S3 suggests that the uplift in diagnoses surrounding the timing of the campaign began to return to pre-campaign levels from around August.

#### Stage at diagnosis

A stage shift for NSCLC patients was seen following the campaign launch (Table 6). When excluding unknowns, the proportion of NSCLCs diagnosed at stage I increased by 3.1 percentage points during the campaign period, (from 14.1% to 17.3%, *P*<0.001; a proportional increase of 22%), and there was a corresponding 3.5 percentage point decrease in the proportion diagnosed at stage IV (from 52.5% to 49.0%, *P*<0.001; a proportional decrease of 7%). There was no evidence for these changes in the control period (*P*=0.404 and 0.244, respectively). There was a significant fall in the percentage of cases with unknown stage, which may be contributing to the change in stage distribution (from 9.5% to 7.6%, *P*<0.001; a proportional decrease of 20%). However, when unknowns were included in the proportions, there was still a significant increase in cases diagnosed at stage I and a significant decrease for stage IV for the campaign period, but not for the control period.

These results add strength to similar trends initially seen for the regional campaign (if excluding unknowns), which did not reach significance (Supplementary Table S8).

The proportion of NSCLC cases diagnosed at stage I appeared to be higher for several months after the campaign (until October) in comparison with the trend for the previous 2 years (Supplementary Figure S4).

The proportion of SCLC patients diagnosed at a limited stage increased non-significantly by 3.0 percentage points for the campaign period (from 28.8% to 31.8%, *P*=0.166) and by 1.7 percentage points for the control period (from 28.7% to 30.4%, *P*=0.440) when excluding unknowns. Including the unknowns also showed non-significant increases (Table 6).

However, following the launch of the regional campaign, there was a significant increase in the proportion of SCLCs diagnosed at limited stage in the pilot area compared with the same time in the previous year (with and without exclusion of unknowns) which did not occur in the control area (Supplementary Table S8).

#### First definitive treatment

A greater proportion of lung cancer patients received surgical resection as a first definitive treatment following the campaign launch (Table 7). The proportion increased by 2.3 percentage points for the campaign period (from 13.7% to 16.0%, *P*<0.001; a proportional increase of 17.0%), with no evidence of a change for the control period (*P*=0.425).

This finding replicated the results seen for the regional pilot campaign (Supplementary Table S9). The proportion receiving surgical resection appeared to return to pre-campaign trends by around September (Supplementary Figure S5).

#### Performance status

The impact of the national campaign on performance status distribution is unclear. The proportion of patients diagnosed with lung cancer with performance status 0 or 1 did not change for the campaign period (an increase of 1.5 percentage points that did not reach statistical significance; *P*=0.075), while the proportion significantly decreased by 2.0 percentage points for the control period (*P*=0.019) (Table 8). The longer-term trends do not suggest a clear pattern associated with the campaign period (see Supplementary Figure S6).

The results for the regional campaign are provided in Supplementary Table S10.

#### NLCA source of referral

For the campaign period, there was an increased proportion of patients diagnosed via GP referral (3.0 percentage point increase from 47.9% to 50.9% *P*<0.001) and a decreased proportion diagnosed after an emergency admission or A&E attendance (1.9 percentage point decrease, from 21.5% to 19.6% *P*=0.004) (Supplementary Table S11). Neither proportion significantly changed for the control (*P*=0.583 and *P*=0.167).

For the regional campaign, changes in the above proportions did not reach significance in either area (Supplementary Table S12).

#### One-year survival

There were similar increases (*P*=0.425) in age-standardised 1-year crude survival between the pilot and control areas for the regional campaign, 4.0 percentage point increase (from 35.2% to 39.2% *P*=0.024) and 2.0 percentage point increase (from 37.3% to 39.3% *P*=0.034), respectively (data not shown).

## Discussion

We believe that the data presented here are the first to suggest a shift to earlier stage lung cancer at diagnosis resulting from a public awareness campaign. There is also evidence to suggest a ‘whole system' response to the campaign from an increase in public awareness and GP attendance, through to more urgent GP referrals and radiological tests. The stage shift was accompanied by a significant increase in the surgical resection rate. Surgery is the main intervention that can lead to improvement in long-term survival of lung cancer and although there are no randomised trials comparing surgery *vs* no surgery in lung cancer (Treasure *et al*, 2012), there is evidence that higher resection rates are associated with a reduced risk of death at a population level (Riaz *et al*, 2012). Therefore, it is reasonable to expect that a sustained increase in resection rate may lead to improved long-term survival and has the potential to be translated into a reduction in lung cancer-specific mortality. However, the PLCO study showed a stage shift in response to CXR screening, but failed to show any significant effect on mortality reduction (Oken *et al*, 2011). We observed a four percentage point improvement in 1-year survival in the intervention area of the regional pilot campaign; however, this was statistically similar to the observed two percentage point increase seen in the ‘control' areas. The impact of the potential shift in referral route from emergency to elective would be expected to impact on the proportion of patients surviving a year (Elliss-Brookes *et al*, 2012).

It should be stressed, however, that these were observational studies and while we have made every effort to identify comparator populations, these could not strictly be described as controls. We cannot therefore claim proof of a causative relationship. However, we believe the case is strong, in particular because data from multiple sources across the patient pathway show trends consistently in the same, potentially beneficial, direction and there were similar trends for both the regional and national versions of the campaign. It should be noted that for some of the metrics there is considerable variation in the data over time, for instance performance status and source of referral (Supplementary Figures S6 and S7), although changes in these metrics are statistically significant, there are a large number of significance tests in this paper and it is possible that despite reaching statistical significance some of these changes may be due to chance alone. Additional time series analysis could further assess the campaigns' impact.

The data we have do not allow us to explain the increased number of cases of lung cancer diagnosed during the intervention period. Our interpretation is that the campaign simply caused more rapid identification and referral of incident cases that already existed and that they were therefore simply diagnosed at an earlier stage. If so, this surge of cases would fall back to a ‘steady state' with the expectation that, if awareness efforts were reinforced and effective over time, the stage shift would also be maintained. However, it is not possible to exclude the over-diagnosis of indolent cancers that would otherwise not have been diagnosed or resulted in death. We think this much less likely to be a significant issue in this symptomatic setting as compared with patients diagnosed in screening programmes of asymptomatic populations. To rule this out, further work would be required, examining detailed characteristics of the tumours diagnosed.

Additionally, lead time bias (the length of time since diagnosis added by detecting disease before its typical clinical presentation that does not affect the disease outcome) is a possible confounder, though likely to be less significant than in the case of screening programmes of asymptomatic patients. The eventual impact of these initiatives will only be demonstrated if there is a fall in disease-specific mortality rate.

Although there was an increase in patient attendance at GP practices, an increase in urgent referrals, and in the numbers of CXRs and CTs carried out, the burden on the NHS appears to have been modest. In particular, anecdotal concerns of GPs being overburdened were perhaps not borne out, with only six additional attendances for patients with a cough per average practice per week and peaks in attendance for cough not reaching those seen during winter (Supplementary Figure S1). However, assessing the cost-effectiveness of such interventions is important and work is being carried out by researchers at the Centre for Health Economics, University of York to model this.

A specific limitation of the results presented is that radiological test data were only available from the month before the campaign launch, so the impact of the campaign on GP-referred diagnostic tests is of limited certainty.

Another limitation of our study is that we have not examined the potential negative impact of the campaigns in any detail. In future evaluations, it will be important to try and assess issues such as unnecessary investigations and interventions in patients who turn out to have benign conditions, while also considering additional benefits of the campaign such as earlier identification of other respiratory diseases.

Campaign duration (5 and nearly 8 weeks) was relatively short and one could hypothesise there may be a cumulative effect over time if similar interventions were repeated. As has been the case for breast cancer awareness over many years (Blanchard *et al*, 2002; Clarke, 2004; Thackeray *et al*, 2013), it is possible that the messages of initiatives such as the ‘Be Clear on Cancer' cough campaign would, if they were sustained over time, begin to ‘spill out' into the wider media to enhance their impact. Initial results for the regional pilot area suggest awareness levels start to fall after rising in relation to the first campaign and repeating the campaign may serve to bring awareness levels back up to where they initially peaked (though not higher).

Such campaigns not only raise awareness in the public, but also in the primary care community. Examining the extent and impact of changes in GP awareness or behaviour on earlier diagnosis is also important. It is not currently possible with these data to disentangle which elements of the intervention were the most successful or whether it is due to the combination, but it would make for interesting and important further research.

Public symptom awareness campaigns for other cancers have also taken place under the Be Clear on Cancer brand, initially under the leadership of DH, and more latterly involving a partnership between DH, Public Health England and NHS England. All the regional and national campaigns, and most of the local-level campaigns, are evaluated using the same metrics, allowing the impact of each campaign to be individually assessed. Due to data availability, the set of data spanning metrics across the pathway is currently less complete for other campaigns compared with the lung cancer campaigns. As more data become available, comparison of results across campaigns is important to learn as much as possible from the significant investment on public awareness raising activity. However, due to numerous differences including tumour type, the nature of symptoms and the clinical pathway, caution should be made when drawing these comparisons. In the public awareness surveys, there were small decreases in spontaneous mentions of some lung cancer symptoms that were not the focus of the campaign. This may reflect a shift in the type of symptoms that come to the forefront of the respondents' minds and/or a shift in the relative significance of symptoms to the respondent. It is reassuring that there were no decreases in prompted awareness of the same symptoms. However, future evaluations of awareness interventions should monitor for the potential impact that only focussing on select symptoms could negatively change the public's perceived significance of other symptoms.

In summary we believe we have, for the first time, demonstrated evidence suggestive of a significant and positive effect of public awareness campaigns promoting the earlier diagnosis of lung cancer, resulting in a stage shift and a higher surgical resection rate. A direct causal link between the campaign and this outcome is impossible to prove, but seems likely given the positive changes along the whole care pathway. Further work on the long-term impact on survival and mortality together with the assessment of their cost-effectiveness is important.

## Figures and Tables

**Table 1:** National campaign: public awareness pre- and post-campaign survey results

**Survey question**	**Pre** ***N*** **(%)**	**Post** ***N*** **(%)**	***P*****-value**^a^	**Non-recognisers**	**Campaign recognisers**	***P*****-value**^b^
Weight base	1153	1121	—	235	886	—
**There are many signs and symptoms of lung cancer. Please name as many as you are aware of** *[Spontaneous awareness]*						
Cough/hoarseness	478 (41%)	560 **(50%)**	<0.001	92 (39%)	468 **(53%)**	<0.001
Persistent/prolonged cough	140 (12%)	168 **(15%)**	0.048	21 (9%)	147 **(17%)**	0.004
TOTAL cough	618 (54%)	728 **(65%)**	<0.001	113 (48%)	615 **(69%)**	<0.001
Shortness of breath	239 (21%)	223 (20%)	0.621	39 (17%)	184 (21%)	0.154
Coughing up blood	224 (19%)	179 **(16%)**	0.031	25 (11%)	154 **(17%)**	0.012
Chest pain	97 (8%)	77 (7%)	0.166	12 (5%)	65 (7%)	0.230
Weight loss	95 (8%)	100 (9%)	0.562	11 (5%)	89 **(10%)**	0.010
**How confident are you that you know the signs and symptoms of lung cancer?** *(Those ‘very confident' or ‘fairly confident')*	514 (45%)	571 **(51%)**	0.002	91 (39%)	480 **(54%)**	<0.001
**I am going to list some symptoms that may or may not be warning signs for lung cancer. For each one can you tell me the extent to which you think it is a warning sign for lung cancer** *(those saying definitely a warning sign) [Prompted awareness]*						
A cough for 3 weeks or more that does not go away	206 (18%)	373 **(33%)**	<0.001	45 (19%)	328 **(37%)**	<0.001
Breathlessness	256 (22%)	278 (25%)	0.144	45 (19%)	233 **(26%)**	0.024
Coughing up blood	619 (54%)	637 (57%)	0.132	109 (46%)	529 **(60%)**	<0.001
A persistent pain in your chest or shoulder	147 (13%)	155 (14%)	0.449	21 (9%)	134 **(15%)**	0.015
Losing weight for no obvious reason	264 (23%)	315 **(28%)**	0.004	45 (19%)	270 **(30%)**	<0.001
A cough that has got worse or changes	231 (20%)	332 **(30%)**	<0.001	46 (20%)	286 **(32%)**	<0.001

Results in bold indicate that the proportion is statistically significantly different to the pre survey proportion or to the proportion for non-recognisers (two-sample test of proportions; *P*<0.05).

a *P*-value for difference in proportions between the pre and post compaign surveys.

b *P*-value for the difference in proportions between non-recognisers and campaign recognisers.

**Table 2:** National campaign: presentations per GP practice per week adjusted for working days excluding bank holidays for patients aged 50+, data from 486 practices

		**Presentations per practice per week (adjusted**^a^)				
**Symptoms**	**Eight-week period**	**2011**	**2012**	**Change (2012** ***vs*** **2011)**	**% Change (2012** ***vs*** **2011)**	***P*****-value**
Key campaign symptom (cough)	Control	6.2	6.5	0.3	**+5**	<0.001
	Campaign	4.8	7.8	3.0	**+63**	<0.001
	Post-campaign	4.1	5.9	1.9	**+46**	<0.001
UTI	Control	1.1	1.1	0.0	+2	0.386
	Campaign	1.1	1.2	0.1	**+5**	0.016
Neck pain	Control	0.8	0.8	0.0	−4	0.142
	Campaign	0.9	0.9	0.0	0	0.963
Shoulder pain	Control	1.9	1.9	0.0	−1	0.604
	Campaign	1.9	1.9	0.0	+1	0.681
Knee pain	Control	2.5	2.5	0.0	+1	0.436
	Campaign	2.8	2.8	0.1	+3	0.060

Control period: 8 weeks from 13th March. Campaign period: 8 weeks from 8th May. Post-campaign period: 8 weeks from 3rd July.

Results in bold indicate a statistically significant change between 2011 and 2012 (likelihood ratio test of two counts; *P*<0.05).

a Adjusted for a 5-day working week excluding bank holidays (e.g., excludes Easter, Early May, Spring bank holidays and Queen's Diamond Jubilee bank holiday for 2012).

**Table 3:** National campaign: number of urgent GP referrals and conversion rate for suspected lung cancer for the campaign and control periods

**Control period**					**Campaign period**					
**Month**	**2011**	**2012**	**% Change (adjusted**^a^)	***P*****-value**	**Month**	**2011**	**2012**	**% Change (adjusted**^a^)	***P*****-value**	***P*****-value control** *vs* **campaign**^b^
**Referrals**										
February	3416	3802	**+6.0**	0.015	May	3472	4817	**+26.1**	<0.001	
March	3993	4149	**+8.6**	<0.001	June	3694	4072	**+27.6**	<0.001	—
April	3523	3580	−3.7	0.114	July	3338	4960	**+41.8**	<0.001	—
February–April total	10 932	11 531	**+3.8**	0.006	May–July total	10 504	13 849	**+31.8**	<0.001	<0.001
**Conversion rate**										
February–April average	24.3%	22.3%	**−2.0**^c^	<0.001	May–July average	24.0%	21.5%	**−2.5**^c^	<0.001	0.479

Results in bold indicate a statistically significant change between 2011 and 2012 (likelihood ratio test of two counts for referrals, and two-sample test of proportions for conversion rates; *P*<0.05).

a Adjusted for a 5-day working week excluding bank holidays (e.g., excludes Easter, Early May, Spring bank holidays and Queen's Diamond Jubilee bank holiday for 2012).

b For the interaction term in the Poisson regression model to test the difference in magnitude of change between the control and campaign periods.

c Percentage point change.

**Table 4:** National campaign: number of chest X-rays and chest with/without abdomen CT scans, May-July compared with April 2012 and 2013^a^

		**Per day**			**Per working day**		
	**No. of tests**	**Tests/organisation**^b^**/day**	**% Change from April**	***P*****-value**	**Tests/organisation/working day**^c^	**% Change from April**	***P*****-value**
**GP-referred chest X-rays**							
**2012**							
April	144 140	25.6	N/A	—	40.4	N/A	—
May–July	566 045	32.8	**+28.3**	<0.001	47.9	**+18.6**	<0.001
**2013**							
April	160 865	32.3	N/A	—	46.1	N/A	—
May–July	404 115	26.7	**−17.3**	<0.001	38.4	**−16.7**	<0.001
**All chest X-rays**							
**2012**							
April	606 840	107.6	N/A	—	169.9	N/A	—
May–July	1 993 105	115.4	**+7.3**	<0.001	168.6	**−0.8**	<0.001
**2013**							
April	600 385	120.6	N/A	—	172.2	N/A	—
May–July	1 624 980	107.5	**−10.8**	<0.001	154.5	**−10.3**	<0.001
**GP-referred chest +/− abdomen CT scans**							
**2012**							
April	2145	0.4	N/A	—	0.6	N/A	—
May–July	8215	0.5	**+25.1**	<0.001	0.7	**+15.7**	<0.001
**2013**							
April	2940	0.6	N/A	—	0.8	N/A	—
May–July	8360	0.6	**−6.3**	0.014	0.8	**−5.8**	0.025
**All chest +/− abdomen CT scans**							
**2012**							
April	28 650	5.1	N/A	—	8.0	N/A	—
May–July	98 455	5.7	**+12.3**	<0.001	8.3	**+3.8**	<0.001
**2013**							
April	31 515	6.3	N/A	—	9.0	N/A	—
May–July	92 580	6.1	**−3.2**	<0.001	8.8	**−2.6**	<0.001

Results in bold indicate a statistically significant change compared with April (likelihood ratio test of two counts; *P*<0.05).

a Data for 2013 are interim data (finalised data were not available at the time of analysis).

b Mostly NHS trusts, but also includes independent organisations too. Numbers of organisations submitting data each period are included in the Supplementary Results.

c A 5-day working week excluding bank holidays (e.g., excludes Easter, Early May, Spring bank holidays, and Queen's Diamond Jubilee bank holiday). The difference before and after the working days adjustment for 2012 is, in part, due to the large proportion of non-working days in April compared with May–July.

**Table 5:** National campaign: number of lung cancers diagnosed for the campaign and control periods

**Control period**				**Campaign period**			
**No. diagnosed**				**No. diagnosed**			
**February**–**April 2011**	**February**–**April 2012**	**% Change (adjusted**^a^**)**	***P*****-value**	**May**–**July 2011**	**May**–**July 2012**	**% Change (adjusted**^a^)	***P*****-value**
7404	7636	+1.5	0.373	7639	8335	**+9.1**	<0.001

Results in bold indicate a statistically significant change between 2011 and 2012 (likelihood ratio test of two counts; *P*<0.05).

a Adjusted for a 5-day working week excluding bank holidays (e.g., excludes Easter, Early May, Spring bank holidays, and Queen's Diamond Jubilee bank holiday for 2012).

**Table 6:** National campaign: number and proportion of non-small cell lung cancers (NSCLC) and small cell lung cancers (SCLC) diagnosed at each stage for the campaign and control periods

		**Control period**				**Campaign period**			
**Stage**		**Feb**–**Apr 2011**	**Feb**–**Apr 2012**	**Change in proportion**	***P*****-value**	**May**–**July 2011**	**May**–**July 2012**	**Change in proportion**	***P*****-value**
**NSCLC**									
I	No. of cases	886	988	—	—	862	1180	—	—
	% Total known	15.2%	15.8%	+0.6	0.404	14.1%	17.3%	**+3.1**	<0.001
	% Grand total	13.6%	14.6%	+1.0	0.082	12.8%	16.0%	**+3.2**	<0.001
									
II	No. of cases	*509*	*593*	—	—	*562*	*660*	—	—
	% Total known	*8.8%*	*9.5%*	+0.7	0.169	*9.2%*	*9.7%*	+0.4	0.397
	% Grand total	*7.8%*	8.8%	**+1.0**	0.041	*8.3%*	*8.9%*	+0.6	0.222
									
IIIA	No. of cases	*857*	*902*	—	—	*859*	*921*	—	—
	% Total known	14.7%	14.4%	−0.3	0.614	14.1%	13.5%	−0.6	0.309
	% Grand total	13.1%	13.3%	+0.2	0.707	12.8%	12.5%	−0.3	0.587
									
IIIB	No. of cases	603	656	—	—	608	720	—	—
	% Total known	*10.4%*	*10.5%*	+0.1	0.840	*10.0%*	*10.5%*	+0.6	0.295
	% Grand total	*9.2%*	*9.7%*	+0.5	0.354	*9.0%*	*9.7%*	+0.7	0.151
									
IV	No. of cases	*2958*	*3117*	—	—	*3201*	*3350*	—	—
	% Total known	*50.9%*	*49.8%*	−1.1	0.244	*52.5%*	*49.0%*	**−3.5**	<0.001
	% Grand total	*45.3%*	*46.1%*	+0.8	0.344	*47.5%*	*45.3%*	**−2.2**	0.008
									
Unknown—uncertain	No. of cases	59	46	—	—	43	50	—	—
	% Grand total	0.9%	0.7%	−0.2	0.147	0.6%	0.7%	0.0	0.783
									
Unknown—not recorded	No. of cases	656	455	—	—	597	513		
	% Grand total	10.0%	6.7%	**−3.3**	<0.001	8.9%	6.9%	**−1.9**	<0.001
									
Total known (stage I–IV)	No. of cases	*5813*	*6256*	—	—	*6092*	*6831*	—	—
	% Grand total	*89.0%*	*92.6%*	**+3.5**	<0.001	*90.5%*	*92.4%*	**+1.9**	<0.001
									
Total unknown	No. of cases	715	501	—	—	640	563	—	—
	% Grand total	11.0%	7.4%	**−3.5**	<0.001	9.5%	7.6%	**−1.9**	<0.001
Grand total		6528	6757	—	—	6732	7394	—	—
**SCLC**									
SCLC—Limited	No. of cases	233	255	—	—	246	286	—	—
	% Total known	*28.7%*	*30.4%*	+1.7	0.440	*28.8%*	*31.8%*	+3.0	0.166
	% Grand total	26.6%	29.0%	+2.4	0.260	27.1%	30.4%	+3.3	0.121
									
SCLC—Extensive	No. of cases	580	584	—	—	609	613	—	—
	% Total known	*71.3%*	*69.6%*	−1.7	0.440	*71.2%*	*68.2%*	−3.0	0.166
	% Grand total	66.2%	66.4%	+0.2	0.919	67.1%	65.1%	−2.0	0.364
									
Unknown—uncertain	No. of cases	10	5	—	—	3	8	—	—
	% Grand total	1.1%	0.6%	−0.6	0.193	0.3%	0.9%	+0.5	0.147
									
Unknown—not recorded	No. of cases	53	35	—	—	49	34	—	—
	% Grand total	6.1%	4.0%	**−2.1**	0.047	5.4%	3.6%	−1.8	0.063
									
Total known	No. of cases	813	839	—	—	855	899	—	—
	% Grand total	92.8%	95.4%	**+2.6**	0.019	94.3%	95.5%	+1.3	0.214
									
Total unknown	No. of cases	63	40	—	—	52	42	—	—
	% Grand total	7.2%	4.6%	**−2.6**	0.019	5.7%	4.5%	−1.3	0.214
Grand total		876	879	—	—	907	941	—	—

Results in bold indicate a statistically significant change between 2011 and 2012 (two-sample test of proportions; *P*<0.05).

**Table 7:** National campaign: first definitive treatment received for lung cancer for the campaign and control periods

	**Control period**				**Campaign period**			
**First definitive treatment**^a^	**No. of cases (% of all cases)**				**No. of cases (% of all cases)**			
	**February**–**April 2011**	**February**–**April 2012**	**Change in proportion**	***P*****-value**	**May–July 2011**	**May–July 2012**	**Change in proportion**	***P*****-value**
Surgery	1076 (14.5%)	1145 (15.0%)	+0.5	0.425	1043 (13.7%)	1331 (16.0%)	**+2.3**	<0.001
Chemotherapy	1909 (25.8%)	1930 (25.3%)	−0.5	0.475	1875 (24.5%)	2150 (25.8%)	+1.2	0.069
Radiotherapy	1366 (18.4%)	1354 (17.7%)	−0.7	0.253	1410 (18.5%)	1462 (17.5%)	−0.9	0.132
Palliative care/active monitoring	2469 (33.3%)	2709 (35.5%)	**+2.2**	0.006	2661 (34.8%)	2817 (33.8%)	−1.0	0.168
Any treatment^b^	6752 (91.2%)	7014 (91.9%)	+0.7	0.146	6890 (90.2%)	7675 (92.1%)	**+1.9**	<0.001
No treatment^c^	652 (8.8%)	622 (8.1%)	−0.7	0.146	749 (9.8%)	660 (7.9%)	**−1.9**	<0.001
Total	7404 (100%)	7636 (100%)	—	—	7639 (100%)	8335 (100%)	—	—

Results in bold indicate a statistically significant change between 2011 and 2012 (two-sample test of proportions; *P*<0.05).

a Patients are counted more than once if they have multiple treatment types on the same earliest treatment date.

b Number of patients receiving any treatment (surgery, chemotherapy, radiotherapy, palliative care, or active monitoring).

c Patients with no treatment type recorded in the database. However, some patients may have had treatment that has not been recorded.

**Table 8:** National campaign: performance status (PS) of lung cancer patients for the campaign months and control periods

	**Control period**				**Campaign period**			
	**No. of patients (% of known)**				**No. of patients (% of known)**			
**PS group**	**February**–**April 2011**	**February**–**April 2012**	**Change in proportion**	***P*****-value**	**May**–**July 2011**	**May**–**July 2012**	**Change in proportion**	***P*****-value**
0 and 1	3576 (54.9%)	3686 (52.9%)	**−2.0**	0.019	3730 (54.8%)	4283 (56.3%)	+1.5	0.075
2	1342 (20.6%)	1451 (20.8%)	+0.2	0.758	1339 (19.7%)	1451 (19.1%)	−0.6	0.358
3 and 4	1594 (24.5%)	1831 (26.3%)	**+1.8**	0.017	1739 (25.5%)	1878 (24.7%)	−0.9	0.228
Total known (0–4)	6512 (100%)	6968 (100%)	—	—	6808 (100%)	7612 (100%)	—	—
Unknown (% of total)	892 (12.0%)	667 (8.7%)	**−3.3**	<0.001	831 (10.9%)	723 (8.7%)	**−2.2**	<0.001
Total	7404	7636	—	—	7639	8335	—	—

PS is measured on a scale of 0–4, where 0 represents patients with normal ability to carry out activities and 4 denotes patients who are completely disabled (confined to a bed/chair).

Results in bold indicate a statistically significant change between 2011 and 2012 (two-sample test of proportions; *P*<0.05).

## Notes

Cancer Research UK as an organisation and MDP have been involved in the development and leadership of the National Awareness and Early Diagnosis Initiative which oversaw the development of the public awareness campaigns.
