# Supplementary material for: An evaluation of the impact of large-scale interventions to raise public awareness of a lung cancer symptom
Source: Br J Cancer. 2014 Dec 2;112(1):207–16. doi: 10.1038/bjc.2014.596 (PMC4453621; doi:10.1038/bjc.2014.596)
Supplement: Supplementary Materials and Methods [file bjc2014596x1.docx]

**Supplementary Materials and Methods**

Details on campaign materials, plus further details of data collection and analysis for the national campaign and details for regional campaign are provided here.

**Development of the Be Clear on Cancer brand and campaigns**

Since 2009/10 the partners of the National Awareness and Early Diagnosis Initiative (NAEDI) have been looking at some of the barriers and motivations that either delay or encourage people to seek help when they have a suspected cancer symptom. Early research reported in April 2010 (Research Works *et al*, 2010) included paired in-depth interviews and focus groups with the target audience, as well as in-depth interviews with GPs, looking at the various audience segments and applied the Department of Health’s Healthy Foundations segmentation model (Department of Health, 2008). Insight from this research, along with the results from other social marketing initiatives and theoretical models, e.g. the Health Belief Model (Becker, 1974 and Rosenstock, 1974), led to the development of the Be Clear on Cancer campaigns.

A number of design concepts were tested with the target audience of C2DE (where C2DE refers to the skilled manual workers, semi & unskilled manual workers and casual or lowest grade workers and those that rely on welfare, including students (National Readership Survey, 2014)) to measure their response. In particular, testing asked if the advertising was relevant to them, if the message was clear and likely to prompt action if they had a symptom. The Be Clear on Cancer brand, developed by M&C Saatchi (2014), came out strongest in the testing and was chosen in 2010 to become the umbrella brand for the Department of Health’s awareness raising campaigns. It was felt this brand would resonate with the target audience and address some of the barriers identified in the early insight work, such as: worry about bothering the GP; not recognising the symptom was serious or could be a sign of cancer; and fear that treatment was worse than death.

At the start of each new campaign, Department of Health and Public Health England engage with a group of experts, including patients, surgeons, GPs and academics. Creative concepts are developed and messages are checked with the expert group to ensure accuracy. Through qualitative research the concepts and key messages are also tested with the target audience for the campaign.

Quantitative research is conducted pre and post regional pilots to assess the impact of the campaign on levels of awareness and to ensure the target audience is responding   to the key messages. In addition, after the regional campaign has finished further qualitative work may be undertaken at this stage if required. If any changes are required to the creative concepts, this will happen prior to the national roll out.

For the lung cancer campaign specifically, early insight work informed the choice not to include any mention of smoking. In addition, following the regional pilot and the quantitative survey conducted pre- and post-campaign, an additional element was added to the lung cancer materials – one that targeted ‘influencers’ such as friends and family, asking them to nudge/prompt someone they may know who has had a persistent cough to go and see their GP. This was applied to radio adverts and posters.

**Lung cancer campaign materials:**

The lung cancer awareness campaign was primarily promoted through two 30 second TV adverts - one targeting a male C2DE population and the other a female C2DE population. Radio and press adverts also ran, along with face to face events in shopping centres and garden centres. Event areas were selected using Mosaic UK profiling (Experian, 2014) and were chosen based on areas where there was likely to be a large number of the target audience. Materials distributed at events included branded symptoms cards, campaign leaflets and referral cards. In addition, leaflets were distributed in surgeries and pharmacies and, during the national campaign, they were used for a door drop in post code areas in Yorkshire which had been identified as having a high proportion of residents of socio-economic groups C2DE aged over 50. Clinicians in both primary and secondary care were informed of the campaign timing, communication activities, aims, message, and target groups, with the aim to prepare them for the potential increase in patient presentations.

**Data collection and analysis**

The Department of Health commissioned Cancer Research UK to carry out the evaluation of the campaigns.

1. **Public awareness and perceived impact on GPs**

**Public awareness surveys**

The Department of Health commissioned TNS BMRB (2014) to carry out the surveys. The overall purpose of the pre- and post-campaign surveys carried out by TNS BMRB was to evaluate the campaign, assessing campaign performance in terms of: awareness of signs and symptoms; recognition of campaign messages; beliefs and attitudes towards cancer and early diagnosis; self-efficacy related to visiting the GP; and emotional engagement with the advertising.

Questions were added to the TNS BMRB in-home, face-to-face Omnibus survey which is carried out across England. Participants were chosen for the public awareness surveys using random location quota sampling (Crouch and Housden, 2003). Although the target age group of the campaign was 50+, data for the surveys were only collected for the population aged 55+.

**Random location quota sampling**: The sampling technique used in this survey was a tightly controlled form of random location quota sampling developed within legacy BMRB, and is the basis of most consumer surveys which TNS BMRB conducts.

The aim of random location sampling is to eliminate the more unsatisfactory features of quota sampling without incurring the cost and other penalties involved in conducting surveys according to strict probability methods.

One of the principal advantages of probability techniques of sampling is that selection of respondents is taken from the hands of interviewers. In conventional quota sampling, on the other hand, interviewers are given quotas to fill, usually from within specified administrative areas. When, for example, an interviewer is asked to complete a quota of AB respondents (people from the highest socio-economic groups), he/she will tend to go to a part of the district where he/she knows such individuals to be available. AB individuals living in mixed social class areas will have little chance of inclusion. This and similar defects lead to biases which are concealed by superficial agreements between sample profiles and accepted standard statistics.

The principal distinguishing characteristic of random location quota sampling, as operated by TNS BMRB, is that interviewers are given very little choice in the selection of respondents. Respondents are drawn from a small set of homogenous streets, selected with probability proportional to population after stratification by their Acorn characteristics (CACI, 2014) and area. Quotas are set in terms of characteristics which are known to have a bearing on individuals' probabilities of being at home and so available for interview. Rules are given which govern the distribution, spacing and timing of interviews.

For the Omnibus survey, the sample of areas takes as its universe all sample units (groups of Census 2001 Output Areas, on average, 300 households) in Great Britain. Output areas are stratified in the following manner:

1. Standard Area
2. Within Standard Area - by Acorn type
3. Within Standard Area by County and ITV Area

Thus, the design is single stage, using direct selection of appropriate groups of output areas, rather than taking streets at random from larger units such as wards or parishes.

Quotas were set by sex (male, female housewife, female non-housewife); within female housewives, presence of children and working status, and within men, working status in order to ensure a balanced sample of adults within effective contacted addresses. Interviewing assignments were conducted over two days of fieldwork and carried out on weekdays from 2pm–8pm and at the weekend. Interviewers were instructed to leave three doors between each successful interview.

*For the national campaign:*

The pre-campaign survey took place 25^th^ April to 6^th^ May 2012 with 1,412 respondents, and the post-campaign survey was 4^th^ to 15^th^ July 2012 with 1,246 respondents. Data were weighted to be representative of the population: a single cell matrix was used in which the matrix consisted of age (55-64 and 65+) by gender (male and female) by Government Office Region (GOR) using the nine GORs in England. Targets were taken from the BARB (Broadcasters’ Audience Research Board) Establishment Survey 2 Years Ending December 2008.

*For the regional campaign:*

There were some slight differences to the methodology for the regional pilot. Pre-campaign interviewing took place 12^th^ September - 7^th^ October 2011 with 571 respondents in the pilot (intervention) area and 452 in the control area, whilst post-campaign interviewing was 14^th^ November - 11^th^ December 2011 with 536 participants in the pilot area and 451 in the control. The Omnibus survey was supplemented with an ad hoc boost in the pilot area of Central England. Both the ad hoc and the Omnibus surveys had their sample drawn by means of random location quota sampling, making them comparable means of data collection to enable merging of data. In the ad hoc boost in pilot areas, sampling points were selected with a bias towards older C2DE residents over-representing the group because this group was of particular interest so a larger sub-sample was available for a sub-analysis. This was then weighted back to be representative at the analysis stage.

Weighting for the regional pilot took a different approach to the national campaign: the two areas, control and pilot, were weighted separately, using weighting matrices designed for each area. The weighting matrix consisted of age by gender taken from the Office For National Statistics (ONS) Mid 2010 Population Estimates (ONS, 2011). The matrix for the control area also accounted for region, using Incorporated Society of British Advertising (ISBA) regions.

Changes in proportions between pre- and post-campaign surveys were tested using the two-sample test of proportions for both the pilot and control areas. Also, post-campaign survey proportions were compared between the pilot area and control area using the two-sample test of proportions. Statistically significant differences must be interpreted with caution for those measures where the pilot area already had a higher awareness compared to the control area in the pre-campaign surveys.

**GP survey**

For the GP survey, a five to seven minute telephone survey was carried out with GPs selected from the Dendrite (2014) health professional database by TNS BMRB and stratified by area and size of practice. Interviews were completed with only one GP per practice.

*For the national campaign:*

Interviewing occurred 9^th^ April - 4^th^ May 2012 for the pre-campaign survey, and 9^th^ July - 3^rd^ August 2012 for the post-campaign survey, with around 300 GPs interviewed in the pre- and post-stages.

*For the regional pilot*:

Pre-campaign interviews took place with GPs in the pilot and control areas from 19^th^ September - 7^th^ October, and post-campaign interviews were held 21^st^ November - 9^th^ December 2011. In the pilot area, 93 and 102 GPs were interviewed pre- and post-campaign, respectively. In the control area, 107 and 98 were interviewed pre- and post-campaign, respectively.

For questions where GPs were asked to estimate numbers (such as number of patients presenting with a cough in the past couple of months), the mean number estimated by those in the pre-campaign survey was compared to the mean number estimated post-campaign using a t-test with the null hypothesis of no difference between two means.

1. **Presentations to primary care**

Mayden (2014) were commissioned to collect data on numbers of patients presenting to GP practices. Mayden invited GP practices to take part in this aspect of the evaluation. Participating practices were asked to provide a data extract for specified criteria. Practice system users were provided with a process document to follow to run a ‘search’ for selecting the data meeting the criteria to ensure a consistent approach. Practices provided data for the specified group of Read codes relating to the key campaign symptom (a cough) as well as certain control symptoms.

*For the national campaign:*

Data were aggregated from 486 GP practices for patients visiting their GP between 1^st^ March 2010 and 31^st^ May 2013. For the analysis, the data were pooled for the campaign period for the eight weeks (the length of the campaign to the nearest whole number of weeks) starting 8^th^ May 2012, the control period as the eight weeks starting 13^th^ March 2012, and the post-campaign period as the eight weeks starting 3^rd^ July, and their corresponding weeks in 2011. The selected control symptoms were urinary tract infection (UTI), neck pain, shoulder pain and knee pain. For each symptom, data were provided for those aged 50 and over, whilst a breakdown was provided for all age groups for cough.

Data were adjusted for working days by dividing each eight week period in 2012 by the number of working days (assuming a five day week excluding bank holidays) in that period and multiplying it by the number of working days for the same period in 2011.

*For the regional pilot:*

Data were extracted from 35 practices across six (formerly existing) Cancer Networks in the pilot area. The specified group of Read codes relating to the key campaign symptom (a cough) also included suspected lung cancer for the regional campaign. It was found that the Read code of suspected lung cancer was rarely used by GPs (less than 1% of Read codes for a cough or suspected lung cancer) and it was decided not to include it for the national campaign evaluation. Data were pooled across the practices for patients visiting their GP between 1^st^ July 2010 and 31^st^ December 2011. A week by week profile of activity (Supplementary Figure S8) showed that there was an increase in GP attendance for cough (and suspected lung cancer) for the five weeks of the regional campaign, and the increase continued for a further three weeks, before returning to a level comparable with the previous year. Therefore, Mayden provided data for the eight weeks following the campaign launch (10^th^ October - 4^th^ December 2011), in comparison to the same weeks in 2010. The number of working days for 10^th^ October - 4^th^ December was the same in 2010 as in 2011, so adjustment for working days was not necessary. Supplementary Figure S9 shows that activity increased for all patients over the age of 30 excluding those aged 85 and over. Therefore, Mayden decided to provide data for attendances for patients aged 30 and over for the key campaign and control symptoms. They also provided a breakdown for all age groups for the key campaign symptom (from which attendances for key campaign symptoms for those aged 50+ could be calculated).

The control symptoms which Mayden selected for the regional campaign evaluation were different to the national campaign. They were migraine, headache, depression and depressed and they were aggregated together.

1. **Urgent GP referrals for suspected lung cancer**

Urgent GP referrals for suspected lung cancer come under the ‘Two Week Wait’ policy (Department of Health, 1997).

*For the national campaign:*

The variables in the Poisson regression model used to test whether the change in number of referrals for the campaign period was significantly different to the change for the control period were as follows: the number of urgent referrals was the outcome variable, with the period type (campaign/control) and time period (year) included as independent variables. Population size was included as an exposure variable (using mid-year population estimates from ONS (2014), with adjustment for the number of working days in each three-month period). Models with and without an interaction term between time period and period type were compared to assess whether any change in the numbers of referrals was significantly different for the campaign period compared with the control.

*For the regional pilot:*

Data from the 27 Primary Care Trusts (PCTs) identified as in the intervention area were compared to the control; pooled data from all other PCTs in England (124 PCTs). The numbers of urgent referrals made in October-December 2011 were compared with those made October-December 2010. A similar Poisson regression model was used as described for the national campaign, but substituting period type (campaign/control) for area type (pilot or control area) and population sizes for each area were estimated using the average size of a PCT.

1. **Chest x-rays and CT scans**

The Diagnostic Imaging Dataset (DID) collects information about diagnostic imaging tests of NHS patients, which is extracted monthly from local radiology information systems (Health & Social Care Information Centre (HSCIC), 2014).

*For the national campaign:*

The organisations submitting data were mostly NHS trusts but they also included a few independent organisations (for example Care UK, for which data were aggregated across all their sites and they were included as one organisation). During April-July 2012, the number of organisations submitting data per month was fairly consistent, only varying between 187 and 188, whilst for the preliminary data available for April-July 2013 the number of organisations varied more: from between 160 and 169 organisations per month (Supplementary Table S13). At the time of data extraction, data for 2013 were yet to be finalised. This means that since then, more organisations may have submitted data, or data may have been resubmitted from others. Therefore, there is a possibility that the number of procedures/organisations submitting data may have changed following publication of the finalised data.

For the analysis, the number of organisations submitting data for May-July was taken as the average for the three individual months.

The data are on the number of tests being performed within each period and not the number of tests requested. This was done for completeness, as the event “date of test request” is not a mandatory field in the DID, whereas the “date of test” is. A working day assumption was used for GP-referred tests to assume that GPs only make referrals on days that they are open, so the more working days in month, the more chest x-rays/CT scans could potentially be requested.

Data were adjusted for days (all days in the month) and working days (a five day week excluding bank holidays) by dividing the number of tests carried out in May-July by the number of days/working days in that period and multiplying it by the number of days/working days in April, carried out for each year separately.

*For the regional pilot:*

Data from the DID are not available for analysing the impact of the regional campaign because the DID only started collecting data from April 2012.

1. **Presentation, stage at diagnosis and treatment in secondary care**

The National Lung Cancer Audit (NLCA) data were provided by month that patients were first seen for lung cancer. The NLCA definition of date first seen is as follows (HSCIC, 2007):

The date of the patient’s first contact with a member of the lung cancer specialist team:

- date of first outpatient appointment (for lung cancer)
- date of outpatient visit when a diagnosis of (lung) cancer was first considered
- date the patient is first seen by the (lung cancer) specialist team in hospital for within-hospital referrals
- date of first booked diagnostic procedure (for lung cancer) if this precedes the first outpatient appointment
- date seen as an emergency, if the patient was first seen as an emergency (within a lung cancer pathway).

*For the regional pilot:*

Data were provided for all NHS trusts, defined by place first seen within the pilot area (32 trusts) and all trusts outside this area within England (141 trusts).

1. **Number of cases diagnosed**: no further details to add.
2. **Stage at diagnosis:** the data for non-small cell lung cancer also includes lung carcinoid tumours. It is recognised that the TNM seventh edition recommends small cell lung cancer is also staged using the TNM system rather than categorising the disease using site-specific limited/extensive staging (SSS) (Sobin *et al*, 2009). However, the data provided use SSS due to historical NLCA standard methodology. Both TNM and SSS are collected in the database and TNM values can be mapped to SSS but not vice versa, resulting in far superior staging completeness for SSS (including the TNM values mapped to SSS) than TNS values alone.
3. **First definitive treatment:** first definitive treatment data were provided for treatment type(s) recorded on the earliest date of treatment. Note that patients are counted more than once if they have multiple treatment types on the same earliest treatment date. Radiotherapy includes external beam therapy and brachytherapy.
4. **Performance status (PS):** Definitions of performance status (HSCIC, 2007):

- 0: Able to carry out all normal activity without restriction
- 1: Restricted in physically strenuous activity but able to walk and do light work
- 2: Able to walk and capable of all self care but unable to carry out any work. Up and about more than 50% of waking hours
- 3: Capable of only limited self care, confined to bed or chair more than 50% of waking hours
- 4: Completely disabled. Cannot carry out any self care. Totally confined to bed or chair.

1. **NLCA source of referral:** it should be noted that the NLCA definitions of the sources of referral may vary from other data sources. More detailed definitions of the NLCA sources of referral are as follows (HSCIC, 2007):

- **Following emergency admission:** After emergency admission includes all acute admissions via A&E, Medical Admissions Unit, etc
- **Following an A&E attendance:** i.e. an out-patient clinic attendance after an A&E visit
- **Referral from a GP:** Referral from GP for out-patient or other non-emergency referrals
- **Referral from a consultant, other than in an A&E department:** If the diagnosis took place within the screening services, then this code applies.
- **Other source of referral:** includes referrals from private healthcare, self-referral (i.e. the patient was not seen previously by a GP), referrals following a domiciliary visit, dental referrals (General) and Dental Practitioner or Community Dental Service referrals.

**f) One-year survival:** Age-standardised one-year crude survival was calculated using International Cancer Survival Standard weights (Corazziari *et al*, 2004). The Poisson regression model to assess whether the difference in survival was significantly different for the pilot area compared to the control area was as follows: the age-standardised number of patients alive after one year was the outcome variable, with the area type (pilot/control) and time period (year) included as independent variables. The number of lung cancer patients was included as an exposure variable. Models with and without an interaction term between time period and area type were compared to assess whether any change in the numbers of patients alive after one year was different for the pilot area compared to the control area.

For the national campaign, there were delays in the processing of data requests from HSCIC, so data were not available to the authors at the time of writing.

**References**

Becker MH (1974). The Health Belief Model and personal health behaviour. *Health Education Monographs, 2*.

CACI (2014). What is Acorn? <http://acorn.caci.co.uk/> (accessed Feb 2014).

Corazziari I, Quinn M, Capocaccia R (2004). Standard cancer patient population for age standardising survival ratios. *Eur J Cancer* **40**(15):2307-2316, DOI:10.1016/j.ejca.2004.07.002

Crouch S, and Housden M. (2003). Marketing research for managers, 3rd ed. Butterworth Heinemann: Oxford.

Dendrite Clinical Systems Ltd (2014). Dendrite Clinical Systems Ltd’s website homepage. <http://www.e-dendrite.com/> (accessed Feb 2014).

Department of Health (1997). The new NHS. Modern, Dependable. The Stationery Office.

Department of Health (2008). Ambitions for health: a strategic framework for maximising the potential of social marketing and health-related behaviour. Crown.

Experian (2014). Mosaic UK. <http://www.experian.co.uk/marketing-services/products/mosaic-uk.html> (accessed Feb 2014)

Health & Social Care Information Centre (2014). Diagnostic Imaging Dataset. <http://www.hscic.gov.uk/did> (accessed Feb 2014).

Health & Social Care Information Centre (2007). The National Clinical Lung Cancer Audit

(LUCADA) Data Manual, Version 3.1. Available from <http://www.hscic.gov.uk/lung> (accessed Feb 2014).

Mayden (2014). Mayden’s website homepage <http://www.mayden.co.uk/> (accessed Feb 2014).

M&C Saatchi (2014). M&C Saatchi’s website homepage <http://www.mcsaatchi.com/> (accessed Feb 2014).

National Readership Survey (2014). Lifestyle and classification data.

<http://www.nrs.co.uk/nrs-print/lifestyle-and-classification-data/> (accessed Feb 2014).

Office for National Statistics (2014). Population statistics: Annual Mid-year Population Estimates for England and Wales. <http://www.ons.gov.uk/ons/taxonomy/index.html?nscl=Population+Estimates> (accessed Feb 2014).

Research Works Ltd, Cancer Research UK and Department of Health (2010). NAEDI Research Presentation of Findings, April 2010. Available from <http://www.cancerresearchuk.org/prod_consump/groups/cr_common/@nre/@hea/documents/generalcontent/cr_045448.pdf> (accessed Oct 2014)

Rosenstock IM (1974). Historical origins of the Health Belief Model. *Health Education Monographs, 2,* 328-335.

Sobin LH, Gospodarowicz MK, Wittekind C (2009). TNM classification of malignant tumours. 7^th^ edn. Union for International Cancer Control , Wiley-Blackwell: Oxford.

TNS BMRB (2014). TNS BMRB’s website homepage <http://www.tns-bmrb.co.uk/> (accessed Feb 2014).
